# Reference values of Forced Expiratory Volumes and pulmonary flows in 3–6 year children: a cross-sectional study

**DOI:** 10.1186/1465-9921-8-14

**Published:** 2007-02-22

**Authors:** Pavilio Piccioni, Alberto Borraccino, Maria Pia Forneris, Enrica Migliore, Carlo Carena, Elisabetta Bignamini, Stefania Fassio, Giorgio Cordola, Walter Arossa, Massimiliano Bugiani

**Affiliations:** 1 SC Pneumologia CPA ASL 4 Torino – Strada dell'arrivore 25/A – 10154 Torino, Italy; 2 Public Health Department University Of Turin – Via Santena 5bis – 10126 Torino, Italy; 3 SSD Pediatria Osp G Bosco ASL 4 Torino – Piazza del Donatore di Sangue, 3 – 10154 Torino, Italy; 4 SC Pneumologia ASO OIRM S. Anna Torino – Corso Spezia, 60 – 10126 Torino, Italy

## Abstract

**Background:**

The aims of this study were to verify the feasibility of respiratory function tests and to assess their validity in the diagnosis of respiratory disorders in young children.

**Methods:**

We performed spirometry and collected information on health and parents' lifestyle on a sample of 960 children aged 3–6.

**Results:**

The cooperation rate was 95.3%. Among the valid tests, 3 or more acceptable curves were present in 93% of cases. The variability was 5% within subjects in 90.8% of cases in all the parameters. We propose regression equations for FVC (Forced Vital Capacity), FEV1, FEV0.5, FEV0.75 (Forced Expiratory Volume in one second, in half a second and in 3/4 of a second), and for Maximum Expiratory Flows at different lung volume levels (MEF75, 50, 25). All parameters are consistent with the main reference values reported in literature. The discriminating ability of respiratory parameters versus symptoms always shows a high specificity (>95%) and a low sensitivity (<20%) with the highest OR (10.55; IC95% 4.42–25.19) for MEF75. The ability of FEV0.75 to predict FEV1 was higher than that of FEV0.50: FEV0.75 predicts FEV1 with a determination coefficient of 0.95.

**Conclusion:**

Our study confirms the feasibility of spirometry in young children; however some of the current standards are not well suited to this age group. Moreover, in this restricted age group the various reference values have similar behaviour.

## Background

Lung disorders in children are quite common[1,2] and usually of an obstructive nature, generally confined to the intra-thoracic, intrapulmonary airways[3]. Reliable information on pulmonary function would aid the diagnostic process and patients' follow up. Studies on respiratory function tests concerning school children and adolescents have already been published [4,5]; and specific criteria for acceptance of maximal expiratory flow volume (MEFV) curves have also been proposed [5]. In recent times attention to this issue in pre-school children has increased [3,6]; different methods and means of measurement have been suggested, particularly spirometry [5,7,8], whole body plethysmography [9], interrupter techniques [10,11]. With regard to spirometry, important studies assessing the feasibility and repeatability of the tests in preschool children have been conducted and have moreover demonstrated that many children are able to perform the required manoeuvres [5,7,8,12-15]. The latest guidelines on standardization of spirometry [16] emphasized that, with appropriate coaching, children as young as 5 years of age are often able to perform acceptable spirometry.

Infants and preschool children normally have large airways in relation to their lung volume [15], thus they empty their lungs more rapidly than older children and adults do. Therefore the forced expiratory volume in one second (FEV1) measurement may be difficult to achieve. Even when an FEV1 is available its clinical value remains questionable because it is often roughly equal to the forced vital capacity (FVC). As a consequence the FEV1/FVC ratio results as being over 90% in the majority of the examined children [15]. In this case it may be more appropriate to report forced expiratory volume of 3/4 of a second (FEV0.75), or FEV0.5 (forced expiratory volume of half a second) as a means for distinguishing abnormality in this age group [13,15].

In cooperating subjects parameters from the descending part of a forced expiration manoeuvre have been proposed as useful indicators of airflow limitation [14].

Some studies conducted on healthy young children have proposed reference equations for FVC, FEV1 and for instantaneous expiratory flows [12-14], but only one of them has proposed reference values for FEV 0.5 [13], and none of the studies retrieved seems to have considered FEV0.75; furthermore none of these parameters have yet been validated with regard to FEV1 and disease or respiratory symptoms.

The aims of this study are twofold:

1) to produce reference values for VC, FEV (Forced Expiratory Volumes) at 1, 0.75 and 0.5 of a second, FEVt/FVC ratios and instantaneous expiratory flow (MEF) respectively for 75%, 50%, and 25% of the expired FVC) for children between 3 and 5 years of age;

2) to discuss the accuracy[17] with which different pulmonary function parameters can distinguish between children with and without respiratory symptoms

## Materials and methods

In 2002, in the context of a study regarding the effects of indoor and outdoor pollution on the respiratory tract, we carried out a survey on a sample of children aged 3–6 attending kindergartens in Turin. We selected 20 kindergartens among the ones located in an area of approximately 500 meters from the 20 air pollution monitoring sites.

A standardized questionnaire[18,19] to be filled in by parents was given to all the children. The questionnaire investigates for the presence of a medical diagnosis of asthma and asthma-like symptoms (occurrence of wheezing and whistling when resting and under strenuous activity, shortness of breath, dry cough, phlegm and chest tightness), rhinitis (frequent sneezing, runny or blocked nose not connected with a cold or flu, itchy or watery eyes), any medical treatment and risk factors.

After the parents' prior approval, the spirometries were carried out in the morning during kindergarten activities; the children's height (measured with a stadiometer), weight and body mass index (BMI) (computed as weight/height^2) were also recorded. Spirometry was performed with the turbine based *Masterscope Rotary Jaeger*. None of the children had ever performed spirometry in the past. To perform the spirometry, like in other studies [12,13] the children were gathered into small groups and using playful communication we explained how to carry out the test. All the children tried a forced expiration before doing the real spirometry. The tests were done standing and with a nose clip. All the tests were performed using special incentive software ("blowing out candles software"). For the initial manoeuvres we encouraged the children to focus their attention on the computer screen: then they were invited to look at the operator's face and perform the manoeuvres together, blowing for as long as possible, and stopping at the operator's command.

The volume-time (V-T) and flow-volume (F/V) tracing obtained were visually inspected to assess the results. Children unable to perform any valid expiratory effort were considered to be non cooperating.

For each child a number of 3–6 MEFV curves were recorded within a 10–15 minute interval. Subjects with only one acceptable manoeuvre were not considered in the analysis.

We have considered not acceptable all the manoeuvres with:

a) a sub-maximal expiratory effort in which a peak expiratory flow (PEF) was not clearly determined (i.e. in presence of flat or rounded curves) [12-15], or with slow rise of PEF (top of the curve to the right)[13];

b) evidence of cough or glottis closure [13,15];

c) an expiration time lesser than 0.5 seconds[15];

d) an abrupt end of expiration effort (presence of a sharp drop or cessation in flow from a point in which the flows where >25% of PEF) [12].

Children with reported skeletal anomalies or lung diseases, other than asthma, were also excluded.

Among all the acceptable subject curves, we considered in the analysis the ones with the largest sum of FVC and FEV1, FEV0.75 or FEV0.5 according to the maximum expiratory time obtained [20].

The instantaneous expiratory flow parameters, as well as those for the other parameters, were obtained from the best curve among the 3–6 attempts recorded and only when these were acceptable were they used for the analysis, in particular:

1. for FVC and MEF75-50-25 reference equations we considered only curves without any kind of early termination;

2. for FEV1, FEV0.75 and FEV0.5 reference equations we considered curves with flow expiration time (FET) respectively ≥ 1, ≥ 0.75 and ≥ 0.5 of a second.

### Data analysis

The absolute and the relative frequencies of non-cooperative subjects were computed and reported by sex and age; the effect of age and sex on cooperation was analyzed by means of logistic regression analysis using cooperation as dependent and age and sex and their interaction (if significant at the 5% level) as predictive variables. The relative and absolute frequencies of subjects according to the exclusion criteria and the number of acceptable manoeuvres performed were also computed and reported: each subject could have more than one cause of exclusion. The repeatability[21] of lung function parameters was evaluated by calculating the absolute difference between the largest value and the second largest value of each parameter (FVC, FEV1, FEV0.75, and FEV0.5) expressed as a percentage of the largest observed measure and reported by class (0–5%, 5.1%–10%, >10%); the absolute difference between the two best FEV1 among the satisfactory manoeuvres was also computed and reported by class of ml (0–100, 101–150, >150). All subsequent analyses were performed using only data from subjects satisfying the acceptability criteria for the parameter considered. Reference values were calculated on healthy children; all the subjects with at least one affirmative answer to the following were excluded:

• asthma during lifetime;

• presence of wheezing not connected with colds;

• a dyspnoea attack;

• chest tightening;

• taking drugs for asthma;

• presence of recurrent cough.

We performed a set of linear regression analyses using gender, age, body height, weight and BMI as predictive variables and lung function forced volumes at 1, 0.75, 0.5 seconds and FVC as dependent variables. The best transformation for each variable was selected with the Box-Cox method [22]. The best-fitting regression model was selected according to the likelihood ratio test [22]. Diagnostic tests for outliers and influential cases were performed and checked for consistency, and if inconsistent they were excluded.

In the presence of variables collinearity (i.e. height vs BMI or BMI vs weight) the one causing the smallest deviance reduction, when introduced into the model, was removed [22,23].

The performance of a lung function variable to detect abnormally decreased airway function in symptomatic subjects was assessed by calculating the sensitivity and specificity for each symptom at the fifth percentile of the reference population, corresponding to a one tail Z score of -1.645 of the regression RSE. Cross tabulation was performed by normal/abnormal lung function parameters and by the presence or the absence of symptoms as previously defined. Odds Ratios (OR) of abnormal tests for symptomatic versus asymptomatic subjects were also computed for each lung function parameter in multiple logistic regression analysis, accounting for confounding effects.

We estimated the ability of FEV0.75 and FEV0.5 to predict FEV1 by means of linear regression using FEV1 as dependent and respectively FEV0.5 and FEV0.75 as predictive variables. In the case of perfect prediction we could expect a constant not different from 0 and a regression coefficient of 1.

## Results

### Quality controls

In the 20 kindergartens a total of 1,249 children aged between 3 and 6 were involved in the study, and the parents' informed consent was retrieved for 1,020 children (81.7%). Out of these, 56 children were absent during the days in which tests were carried out and 4 refused to undergo the lung examination. Spirometries were performed on a total of 960 children, with a cooperation rate of 95.3% (45 non cooperative children).

The cooperation rate (Table 1) was significantly higher in children older than 3 years of age (Chi-square 11.68, p < 0.01) with not significant differences between genders (Chi-square 0.37, p > 0.10).

**Table 1:** Number and proportion of non cooperating subjects at the spirometry tests, divided according to age and sex

	Females			Males			Total		
Age (yrs)	Total	Non cooperating		Total	Non cooperating		Total	Non cooperating	
						
		N	%		N	%		N	%

3	20	2	10.0	29	6	20.7	49	8	16.3*
4	204	9	4.4	230	12	5.2	434	21	4.8
5	200	6	3.0	222	8	3.6	422	14	3.3
6	20	1	5.0	35	1	2.8	55	2	3.6
Tot	450	18	4.0	509	27	5.3	960	45	4.7

* χ^2^= 11.68, p < 0.01

Among the 915 cooperative subjects, 149 (16.3%) tests results were excluded as they lacked one or more of the acceptability criteria (Table 2): a total of 766 (83.7%) tests results were included for the analysis.

**Table 2:** Numbers and percentage of excluded subjects by exclusion criteria and number of acceptable tests

Exclusion criteria and acceptable test	N	%
Curves with a sub-maximal expiratory effort	56	6.1
Only 1 acceptable manoeuvre	4	0.4
Early interruption	100	10.9
Expiration time < 0.5 sec	7	0.8

Total excluded subjects	149	16.3
Subjects with acceptable tests	766	83.7

No age nor gender distribution differences were found either in the included or in the excluded subjects (p < 0.05). Three or more acceptable curves, resulting from the validity test, were present in 93% of cases. The frequency of exclusion, among the 3-year old children, was higher but not statistically significant.

The whole group Flow Expiration Time (FET) mean was 1.1 seconds (IC 95% 1.07–1.13) with non significant gender and age variation.

Among the 766 tests included for further analysis, we observed in 278 cases an early termination with the presence of a sharp drop or a cessation in flow from a point in which the flows where <25% of PEF. These 278 subjects were considered only for their FEV_0.5_, FEV_0.75 _and FEV_1 _and not for FVC and flow analysis (see tab3).

The repeatability of FVC and FEVt for all the parameters was fairly good, with a variability = 10% for almost all the children (higher than 99%) and within 5% in 90.8% of cases: the absolute variability among the different manoeuvres of the same subjects was under 100 ml for 98.6% of subjects, and under 150 ml for all subjects. The MEF25, MEF50 and MEF75 repeatability was lower than the volumes repeatability. Among flows, MEF75 had the smallest variability (< = 10% for 84.4% of subjects) (Table 3).

**Table 3:** Repeatability within subject of lung function parameters expressed as within subjects variation coefficient (standard deviation within the two best/mean of the two best%)

	FVC^a^	FEV_1 _^b^	FEV_0.75 _^c^	FEV_0.5 _^d^	MEF_75 _^e^	MEF_50 _^f^	MEF_25 _^g^
N	458	576	680	766	458	458	458
Mean (%)	2.0	2.2	2.2	2.1	6.0	9.1	16.3
Max (%)	12.4	13.6	13.7	14.1	51.9	51.9	92.3
*0–5% (%)*	*90.8*	*90.8*	*92.1*	*90.9*	*65.0*	*41.1*	*23.9*
*6–10% (%)*	*8.1*	*8.5*	*7.5*	*8.4*	*19.4*	*33.5*	*20.2*
*10%+ (%)*	*1.1*	*0.7*	*0.4*	*0.8*	*15.6*	*25.3*	*56.0*

^a^FVC = forced vital capacity;

^b^FEV_1_= forced expiratory volume in one second;

^c^FEV_0.75 _= forced expiratory volume in 3/4 of a second;

^d^FEV_0.5 _= forced expiratory volume in half a second;

^e^MEF_75 _= instantaneous expiratory flow when 25% of FVC has to be expired

^f^MEF_50 _= instantaneous expiratory flow when 50% of FVC has to be expired

^g^MEF_25 _= instantaneous expiratory flow when 75% of FVC has to be expired

### Reference values

Table 4 reports the anthropometric characteristics of the 766 subjects. The girls were slightly taller and heavier than the boys. The 5^th ^and 95^th ^BMI percentile (mean ± 1.64 standard deviations) were respectively 12.6 and 18.8 over all (12.9 and 19.1 for females; 12.4 and 18.4 for males), within the normal range reported in literature for these ages [18,19].

**Table 4:** Characteristics (mean and standard deviation) of the 766 subjects considered for estimation and validation of the reference values

Gender	Age (yrs)	N	Height (cm) m *(sd)*^§^	Weight (kg) m *(sd)*^§^	BMI* m *(sd)*^§^
Males	3	19	104.6 *(3.5)*	16.3 *(1.9)*	14.9 *(1.5)*
	4	181	107.3 *(4.9)*	17.8 *(2.6)*	15.4 *(1.8)*
	5	177	113.3 *(4.6)*	19.9 *(2.8)*	15.4 *(1.8)*
	6	29	118.7 *(5.5)*	22.9 *(4.2)*	16.2 *(2.2)*
	**Total**	**406**	**110.5*(5.9)***	**18.9 *(3.1)***	**15.4 *(1.8)***
Females	3	15	105.1 *(4.4)*	17.9 *(1.8)*	16.2 *(1.2)*
	4	163	108.9 *(4.7)*	18.7 *(2.5)*	15.7 *(1.7)*
	5	162	114.7 *(5.5)*	21.1 *(3.7)*	16.0 *(2.1)*
	6	20	119.3 *(5.7)*	24.3 *(6.0)*	17.0 *(3.3)*
	**Total**	**360**	**112.0 *(5.6)***	**20.1 *(3.0)***	**16.0 *(1.9)***
Whole group	3	34	104.9 *(4.0)*	17.4 *(1.9)*	15.8 *(1.4)*
	4	344	108.2 *(4.9)*	18.3 *(2.6)*	15.6 *(1.8)*
	5	329	114.0 *(5.1)*	20.5 *(3.4)*	15.7 *(2.0)*
	6	49	119.1 *(5.6)*	23.8 *(5.4)*	16.7 *(2.9)*
	**Total**	**766**	**111.3 *(6.2)***	**19.6 *(3.5)***	**15.7*(1.9)***

* BMI = Body Mass Index

^§ ^m (*sd) *= mean (standard deviation)

Table 5 reports the lung function parameter means and standard deviations by symptom status. In asymptomatic subjects the lung function parameter values were slightly higher than those in symptomatic subjects. The Box-Cox test[22] for regression analysis of the lung function parameters versus anthropometric variables showed that no linear transformation of dependent or independent variables was necessary. Among asymptomatic subjects the multiple regression analysis (Table 6), using gender, age, height and BMI as covariates, demonstrated that the static and the dynamic lung volumes were significantly higher in females than in males. A significant age positive effect was detected for all lung volumes except for FVC and FEV1. A significant positive effect was also detected for all lung volumes which was independent from height and BMI. Body weight was collinear (Variance Inflation Factor, VIF >18) with BMI and it was therefore excluded on the basis of the Likelihood Ratio (LR)[22] test (p = 0.09 for weight p = 0.04 for BMI).

**Table 5:** Distribution (mean and standard deviation) of lung function parameter by symptom status

	Asymptomatic		Symptomatic		Total	
	N	m *(sd)*^§^	N	m *(sd)*^§^	N	m *(sd)*^§^
FVC (lt)^a^	327	1.10*(0.22)*	128	1.07*(0.24)*	455	1.09*(0.23)*
FEV_1 _(lt)^b^	409	1.09*(0.20)*	169	1.05*(0.21)*	578	1.08*(0.21)*
FEV_0.75_(lt)^d^	493	1.04*(0.19)*	190	0.98*(0.19)*	683	1.02*(0.19)*
FEV_0.5 _(lt)^c^	562	0.90*(0.16)*	205	0.86*(0.16)*	767	0.89*(0.16)*
FEV_1_/FVC	285	0.96*(0.04)*	116	0.96*(0.04)*	401	0.96*(0.04)*
FEV_0.75_/FVC	311	0.92*(0.05)*	123	0.91*(0.07)*	434	0.92*(0.06)*
FEV _0.5_/FVC	327	0.81*(0.07)*	128	0.80*(0.09)*	455	0.81*(0.08)*
MEF_75_(lt/s)^e^	327	2.32*(0.50)*	128	2.18*(0.53)*	455	2.28*(0.51)*
MEF_50_(lt/s)^f^	327	1.66*(0.38)*	128	1.57*(0.40)*	455	1.64*(0.39)*
MEF_25_(lt/s)^g^	327	0.85*(0.24)*	128	0.82*(0.28)*	455	0.84*(0.25)*

§m(sd) = mean (standard deviation)

^a^FVC = forced vital capacity;

^b^FEV_1_= forced expiratory volume in one second;

^c^FEV_0.75 _= forced expiratory volume in 3/4 of a second;

^d^FEV_0.5 _= forced expiratory volume in half a second;

^e^MEF_75 _= instantaneous expiratory flow when 25% of FVC has to be expired

^f^MEF_50 _= instantaneous expiratory flow when 50% of FVC has to be expired

^g^MEF_25 _= instantaneous expiratory flow when 75% of FVC has to be expired

**Table 6:** Multiple regression coefficients (*β*) of the lung function parameters (*Y*)* versus anthropometric variables (in asymptomatic subjects)(*x*)

Lung function parameters	Sex (males)	Age (years)	Height (cm)	BMI (%)	Weight (Kg)	Costant	N	R^2	RSE
FVC	-0.049^b^	0.018	0.026^c^	0.015^c^	[0.011]	-2.042^c^	328	0.57	0.15
FEV_1_	-0.042^b^	0.038	0.023^c^	0.017^c^	[0.013]	-1.907^c^	409	0.59	0.13
FEV_0.75_	-0.034^c^	0.023^b^	0.022^c^	0.015^b^	[0.008]	-1.729^c^	494	0.59	0.12
FEV_0.5_	-0.031^c^	0.024^b^	0.017^c^	0.011^b^	[0.001]	-1.311^c^	564	0.55	0.11
MEF_75_	0.059	0.108^b^	0.046^c^	0.024^c^	[-0.113]	-3.385^c^	328	0.41	0.39
MEF_50_	0.002	[0.024]	0.033^c^	[0.015]	[-0574]	-2.269^c^	328	0.29	0.32
MEF _25_	0.012	[-0.005]	0.018^c^	[0.075]	[-0.538]	-1.152^c^	328	0.19	0.22

a = p < 0.05; b= p < 0.01; c = p < 0.001;

BMI = body mass index;

*β *= Multiple Regression Coefficient;

FVC = forced vital capacity;

FEV_1_= forced expiratory volume in one second;

FEV_0.75 _= forced expiratory volume in 3/4 of a second;

FEV_0.5 _= forced expiratory volume in half a second;

MEF_75 _= instantaneous expiratory flow when 25% of FVC has to be expired

MEF_50 _= instantaneous expiratory flow when 50% of FVC has to be expired

MEF_25 _= instantaneous expiratory flow when 75% of FVC has to be expired

[ ] brackets variables = excluded by log-likelihood ratio test because collinear or NS and not influent.

* *Reference values were computed as y' = Summ(BX)+constant; the 5*^*th*^percentiles of reference values "as normality limit" was computed by subtracting to y' the 1.64* Root MSE

Among instantaneous maximum expiratory flows, also shown in Table 6, MEF75 increases with age, height and BMI and decreases, but without statistical significance, with weight with a R^2 of 0.41; MEF50 and MEF25 increase significantly only with height. (R^2 = 0.29 for MEF50 and 0.19 for MEF25). Because of the little and not significant effect shown, the age, the weight and the BMI were excluded from the final model.

The regression of volume time, expressed as an FVC fraction, versus anthropometric variables did not show any significant effect: a poor height effect gave a determination coefficient that is lower than 4%. The values were distributed asymmetrically and no linear transformation was able to correct for the absence of normality [22]. The lower normality limits were computed as the 5^th ^percentile of non parametric distribution in asymptomatic children (Table 7).

**Table 7:** Mean and 5^th ^percentile as limit of normal value in asymptomatic subjects

Parameter	N	Mean	5^th^ntil
FEV_1_/FVC	285	0.96	0.88
FEV_0.75_/FVC	311	0.92	0.83
FEV_0.5_/FVC	327	0.81	0.69

FEV_1_/FVC = ratio of forced expiratory volume in one second and forced vital capacity;

FEV_0.75_/FVC = ratio of forced expiratory volume in 3/4 of a second and forced vital capacity;

FEV_0.5_/FVC = ratio of forced expiratory volume in half a second and forced vital capacity;

### Validation of reference values

**Figure 1:**
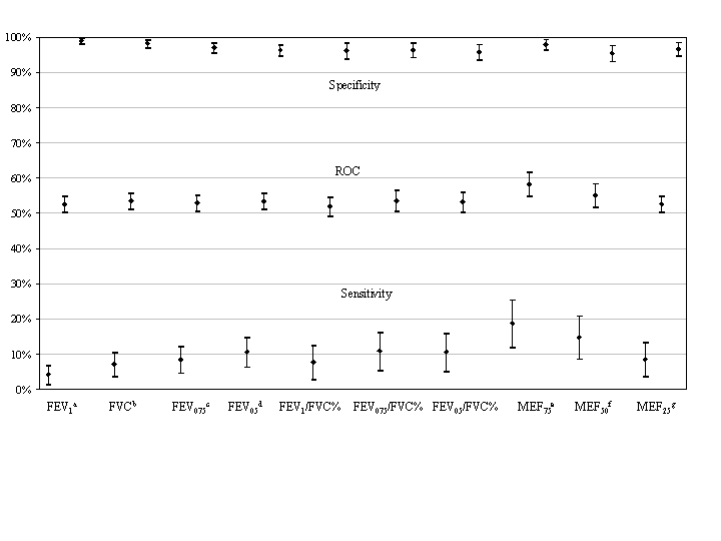
**Sensitivity and specificity (and CI95%) versus symptoms in children of reduced function parameters**. ROC = Receiver Operating Characteristic. ^a^FVC = forced vital capacity; ^b^FEV_1_= forced expiratory volume in one second; ^c^FEV_0.75 _= forced expiratory volume in 3/4 of a second; ^d^FEV_0.5 _= forced expiratory volume in half a second; ^e^MEF_75 _= instantaneous expiratory flow when 25% of FVC has to be expired. ^f^MEF_50 _= instantaneous expiratory flow when 50% of FVC has to be expired. ^g^MEF_25 _= instantaneous expiratory flow when 75% of FVC has to be expired

The test sensitivity and its specificity versus symptoms in children with a reduced function parameter are reported in Figure 1. These were computed as the observed value, lower than predicted, minus 1.64 regression standard error (RSE) for FVC, FEVt and MEFx, and lower than the 5^th ^percentile for FEVt/FVC%.

The sensitivity and specificity are comparable among all the recorded parameters: MEF75 showed the highest sensitivity (18.8%) with a specificity of 97.9% and a combination (ROC Receiver Operating Characteristic) of 58%; FVC showed the worst sensitivity (4.2%) and the best specificity (98.9%).

In Table 8 we reported the strength of association, expressed as OR, between symptom status and lung function normality parameters, adjusted for age, sex and anthropometric variables. Each given parameter, except for the FEV1/FVC% ratio, was significantly associated with symptoms. MEF75 and FEV1 showed the higher association with an OR respectively of 10.6 and 4.2.

**Table 8:** Odds ratios (OR) of lung function parameters lower than 5^th ^percentile of reference value versus symptoms using "list-wise deletion"* of missing values and a single parameter

Parameter	OR	CI 95%	*p *value
FVC	4.05	1.41–11.61	0.009
FEV_1_	4.17	1.85–9.39	0.001
FEV_075_	2.93	1.48–5.82	0.002
FEV_0.5_	3.10	1.66–5.76	0.000
FEV_1_/FVC	2.10	0.84–5.20	0.111
FEV_0.5_/FVC	3.22	1.45–7.18	0.004
FEV_0.75_/FVC	2.71	1.22–6.02	0.015
MEF_75_	10.55	4.42–25.19	0.000
MEF_50_	3.63	1.78–7.38	0.000
MEF_25_	2.70	1.14–6.40	0.024

* The single case is eliminated for the variable in which a missing value is present

FVC = forced vital capacity;

FEV_1 _= forced expiratory volume in one second;

FEV_0.75 _= forced expiratory volume in 3/4 of a second;

FEV_0.5 _= forced expiratory volume in half a second;

MEF_75 _= instantaneous expiratory flow when 25% of FVC has to be expired

MEF_50 _= instantaneous expiratory flow when 50% of FVC has to be expired

MEF_25 _= instantaneous expiratory flow when 75% of FVC has to be expired

### Relationship between volumes/time measurements

The regression equations were calculated using FEV1 as dependent variable and respectively FEV0.75 and FEV0.5 as predictive, and using FEV0.75 as dependent and FEV0.5 as predictive variable (Table 9). The FEV0.75 showed to be suitable to predicts FEV1 quite well, with a 0.95 of determination coefficient: FEV1 is expected to increase by 1.06 litres for each litre of increase in FEV0.75 with an intercept nearly equal to 0. The FEV1 prediction, by using the measured FEV0.5, is less precise than using the FEV0.75 with a 44 millilitres of systematic difference (intercept): FEV1 is expected to increase by 1.166 litres. For every increase of 1 litre in FEV0.5, the determination coefficient resulted to be 0.87.

**Table 9:** Regression equation using FEV_1 _as dependent variable and respectively FEV_0.75 _and FEV_0.5 _as predictive

Parameter	*β*	CI 95%	a	CI 95%	R^2^
FEV_0.75_	1.06	(+1.04; +1.08)	-0.01	(-0.02; +0.02)	0.950
FEV_0.5_	1.17	(+1.13; +1.21)	0.04	(+0.01; +0.08)	0.870

R^2 ^= determination coefficient (proportion of explained variance);

a = intercept or constant and *β *= regression coefficient;

FEV_0.75 _= forced expiratory volume in 3/4 of a second;

FEV_0.5 _= forced expiratory volume in half a second.

## Discussion

### Quality control

In this study spirometries in young children were analyzed to determine whether they met the published quality control criteria and to examine the possible differences.

A more recent guideline[16] has marginally discussed the issues peculiar to spirometric examination in young children and it says that the examination is considered just as feasible in this age group as it is in adults; indices derived from blowing and recording the expiratory times of <1 second were considered to have clinical usefulness. However, the data shown for recommending the use of FEV0.5 and FEV0.75 for clinical purposes were insufficient. Furthermore, in the criteria to evaluate the duration of the test, these guidelines recommend that "*the V-T curve shows no changes for = 1 second and the subject tries to exhale for ≥ 3 seconds in children aged <10 years*"[16], without any additional specification.

This study confirms the feasibility of spirometric examinations in symptomatic or asymptomatic young children, but our results suggest that, because of the too short expiration time, the last guideline indication is not applicable in children younger than 6. The mean FET observed in our children were all around 1 second.

In 3-year-old subjects the cooperation rate was low (83.7%) but high enough to justify the use of spirometry in this age group; in children over 3 years of age the cooperation increases and the success rate becomes comparable to that of other studies based on samples of the general population [7,8,12-14].

A lower success rate was reported in studies conducted on patients with respiratory diseases or in the case of very restrictive exclusion criteria [15]. Moreover, we obtained tests with at least 3 acceptable curves and with a variability among the requested manoeuvres lower than 10% in almost all of the cooperative children.

A critical problem observed in our sample concerns the early termination of many tests; it could be partly explained in relation to the psychomotor maturation of children in which there is an early realization of an equal pressure point at a point less close to the distal airways [4,24-26].

Nevertheless, in many cases early termination may be influenced by methodological or software issues as well[27,28]. A limit of our study is to be discussed in the incentive software used. The candle blowing incentive software produced by Jaeger is a good tool for early training or for encouraging peak flow manoeuvres, but it is less suitable when a full forced expiration is required [15,27]. This problem was addressed and partly limited by using an interactive procedure to perform the test: the children were requested to imitate and reproduce the operator's manoeuvres. Tests with an abrupt cessation of expiration need to be analyzed with caution. Due to lack of consensus on exclusion criteria[12,27,29], the choice of setting a cut-off of 25% of the PEF was done in order to balance the opposing requirements of having the best quality control and recording the largest quantity possible of useful information. Early termination should be quantified and pointed out in the lung function tests reports and, when it occurs, FEVt/FVC and MEFX parameters might not be registered.

With regard to quality control and acceptance criteria, in agreement with other authors [15], a realistic approach could be that of accepting tests with at least 2 curves of maximum effort (PEF easily observable) and with a difference among parameters within 100 ml (10% of FVC in our sample), to exclude tests lasting less than 0.5 seconds and to accept tests with small early interruptions of expiration.

### Reference equations

Using the previously discussed quality control criteria we were able to propose the first reference equation for FEV0.75 (as far as we know) and new reference values for FVC, FEV1, FEV0.5 and for instantaneous expiratory flows (MEF75-MEF50-MEF25) based on a large sample of young children.

The increase of lung volumes with BMI, accounting for height, age and gender, reflects the effect of body size [13]or physical fitness[30,31]; although obesity is reported to determine a reduction of lung function values[32], this is not proved in our sample due to the BMI being within normal ranges in more than 95% of the population studied. Body weight seems to have a less important effect when controlling for the BMI.

The lack of a significant effect of age on FVC is probably due to the small size of the sample with an acceptable FVC measurement and to the limited age range in our study: in any case, we observed an increase in dynamic volumes of 23 ml in FEV1 and of 15 ml in FEV0.75 for each year of age increase.

About the gender effect found in our study, it is known that girls have better physiological performances than males in preadolescence[13,33]: the lack of any significant effect described in other studies on young children is probably the consequence of a lower statistical power[12]. Concerning the validity of reference values, studies conducted on infants[25,26] suggest that flows have a better discriminative power versus symptoms than timed volumes can have. In young children FEVt (particularly FEV0.75 and FEV0.5) are certainly easier to achieve than forced expiratory flows are, furthermore these also have a higher repeatability.

Symptomatic subjects have a more elevated occurrence of functional alterations when compared to asymptomatic ones, nevertheless the test's sensitivity here is far from optimal. In fact, as normally to be expected in the young, children are usually defined as symptomatic on the basis of symptoms in the last 12 months reported by parents in the questionnaires. Hence, measurements often occur in a symptom-free period[13,34,35].

The 95% specificity, for asymptomatic children, is expected by definition using regression methods to calculate reference values. MEF75 and, secondarily, FEV1, versus symptoms, seems to be the parameter with the best sensitivity/specificity combination and the best discriminating ability; the FEV0.75 or the FEV0.5 should be considered to be adequate to be used when FEV1 was not obtained or its validity is under discussion (e.g. because of a too short expiratory time). To confirm the previous sentence it would be necessary to show that, particularly for this age group, the forced volumes expired in a time of shorter than 1 second is able to discriminate between healthy and diseased equally, or even better, than FEV1 is able to do: nevertheless this issue can be better resolved in a well designed case-reference or longitudinal cohort study but this is beyond the scope of the current study design.

The physiological implications of the different timed forced expiratory are as yet not well understood. Similarly to other studies on pulmonary flows in young children we observed that the FEV1 is rarely obtainable and, when retrieved, it is somewhat identical to the FVC. Our results showed that the FEV1, when absent or not reliable, could be estimated from FEV_0.75 _applying the corrections emerged (see Table 6). The corrected FEV_0.75 _could be considered a reliable proxy of the FEV1 to be used in those epidemiological studies in which emerge the need to compare flux parameters results in different age strata.

In conclusion, reproducible spirometry can be obtained in the majority of young children aged between 3 and 6 years old. Performing spirometry and using all measurable parameters in this age group has the potential to improve the assessment and the management of pulmonary diseases. In particular the forced expiratory volumes in less than 1 second may provide useful clinical information. It is recommended that such parameters should be collected in young children performing spirometry and further studied for their physiological and clinical significance.

## Competing interests

The author(s) declare that they have no competing interests.

## Authors' contributions

All of the authors have participated sufficiently in the work to take public responsibility for the whole content of it; in particular the following made substantial contributions to the intellectual content as described below:

PP contributed substantially for the conception and design and the drafting of the manuscript; AB contributed for the critical revision of the manuscript and for important intellectual content; MPF contributed to the drafting of the manuscript; EM contributed in the analysis and interpretation of data; CC contributed to the acquisition of data, technical, and material support; EB contributed to the acquisition of data, technical, and material support; SF contributed to the acquisition of data, technical, and material support; GC contributed to the acquisition of data, technical, and material support; WA contributed obtaining funding, technical, and material support and MB contributed substantially for obtaining funding, the supervision and the critical revision of the manuscript, for important intellectual content

## Grants

This study was supported by a grant from the Region Piedmont, Italy
